# Multimodular fused acetyl–feruloyl esterases from soil and gut Bacteroidetes improve xylanase depolymerization of recalcitrant biomass

**DOI:** 10.1186/s13068-020-01698-9

**Published:** 2020-03-31

**Authors:** Cathleen Kmezik, Cyrielle Bonzom, Lisbeth Olsson, Scott Mazurkewich, Johan Larsbrink

**Affiliations:** 1 Division of Industrial Biotechnology, Department of Biology and Biological Engineering, Chalmers University of Technology, 412 96 Gothenburg, Sweden; 2 Wallenberg Wood Science Center, Chalmers University of Technology, 412 96 Gothenburg, Sweden

**Keywords:** Carbohydrate-active enzyme, Carbohydrate esterase, Multidomain enzymes, Xylan, Polysaccharide utilization locus, Acetyl xylan esterase, Feruloyl esterase, Corn cob, Beech wood

## Abstract

**Background:**

Plant biomass is an abundant and renewable carbon source that is recalcitrant towards both chemical and biochemical degradation. Xylan is the second most abundant polysaccharide in biomass after cellulose, and it possesses a variety of carbohydrate substitutions and non-carbohydrate decorations which can impede enzymatic degradation by glycoside hydrolases. Carbohydrate esterases are able to cleave the ester-linked decorations and thereby improve the accessibility of the xylan backbone to glycoside hydrolases, thus improving the degradation process. Enzymes comprising multiple catalytic glycoside hydrolase domains on the same polypeptide have previously been shown to exhibit intramolecular synergism during degradation of biomass. Similarly, natively fused carbohydrate esterase domains are encoded by certain bacteria, but whether these enzymes can result in similar synergistic boosts in biomass degradation has not previously been evaluated.

**Results:**

Two carbohydrate esterases with similar architectures, each comprising two distinct physically linked catalytic domains from families 1 (CE1) and 6 (CE6), were selected from xylan-targeting polysaccharide utilization loci (PULs) encoded by the Bacteroidetes species *Bacteroides ovatus* and *Flavobacterium johnsoniae*. The full-length enzymes as well as the individual catalytic domains showed activity on a range of synthetic model substrates, corn cob biomass, and Japanese beechwood biomass, with predominant acetyl esterase activity for the N-terminal CE6 domains and feruloyl esterase activity for the C-terminal CE1 domains. Moreover, several of the enzyme constructs were able to substantially boost the performance of a commercially available xylanase on corn cob biomass (close to twofold) and Japanese beechwood biomass (up to 20-fold). Interestingly, a significant improvement in xylanase biomass degradation was observed following addition of the full-length multidomain enzyme from *B. ovatus* versus the addition of its two separated single domains, indicating an intramolecular synergy between the esterase domains. Despite high sequence similarities between the esterase domains from *B. ovatus* and *F. johnsoniae*, their addition to the xylanolytic reaction led to different degradation patterns.

**Conclusion:**

We demonstrated that multidomain carbohydrate esterases, targeting the non-carbohydrate decorations on different xylan polysaccharides, can considerably facilitate glycoside hydrolase-mediated hydrolysis of xylan and xylan-rich biomass. Moreover, we demonstrated for the first time a synergistic effect between the two fused catalytic domains of a multidomain carbohydrate esterase.

## Background

**Fig. 1:**
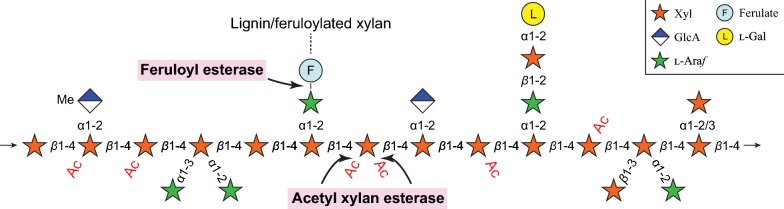
Schematic structure of xylan. The density and types of substitutions on the backbone is highly dependent on the plant species, where hardwood GX (glucuronoxylan) is more sparsely decorated compared to grass AX (arabinoxylan) and GAX (glucuronoarabinoxylan). Feruloyl side groups may link covalently to either lignin or other feruloyl moieties on neighbouring xylan chains. Arrows indicate where feruloyl esterases (FAEs) and acetyl xylan esterases may act, respectively

Plant biomass is an abundant and renewable carbon source [1]. However, the complex and heterogeneous cell wall structure comprising the majority of this resource is very recalcitrant to degradation which hampers enzymatic hydrolysis. Subsequently, industrial processes based on the utilization of sugar from biomass suffer from low productivity and a lack of economic feasibility. Xylan is the second most abundant polysaccharide present in hardwoods and grass species [2]. It consists of a *β*-1,4-linked xylose backbone incorporating a variety of substitutions and non-carbohydrate decorations depending on the plant species and tissue (Fig. 1). In vascular plant cell walls, *α*-1,2-linked 4-*O*-Me-d-glucuronic acid substitutions are ubiquitous (glucuronoxylan; GX), while l-arabinofuranosyl moieties linked at either or both the *α*-1,2 and *α*-1,3 positions are characteristic for arabinoxylan (AX) which is predominant in grass species [3]. The most densely substituted form of xylan is glucuronoarabinoxylan (GAX) which, in addition to the aforementioned substitutions, includes *α*-1,2-, *α*-1,3- or *β*-1,3-xylosyl substitutions and more complex side chains [4]. Non-carbohydrate decorations like *O*-acetylation of xylan is common in eudicots and monocots, on the *O*-2 and/or *O*-3 positions of the xylan backbone [5, 6], but is not found in xylan from conifers [7]. Feruloyl (5-*O*-*trans*-feruloyl-l-arabinofuranosyl) decorations are only present in commelinid monocots (e.g. corn) [8, 9], which can in turn form diferulate esters between neighbouring feruloylated GAX chains, thereby crosslinking the polysaccharides and creating recalcitrant matrices [10]. The amount of ferulic acid in plant biomass can be high, with as much as 3% of the dry weight in corn bran [11]. In some species, other hydroxycinnamic acid decorations have also been found [8]. Previous studies have shown how the presence and quantity of non-carbohydrate decorations on the xylan backbone hamper the action of *endo*-xylanases and need to be addressed for an efficient biomass conversion process [12, 13].

To facilitate metabolism of complex polysaccharides like xylan, bacteria from the phylum Bacteroidetes have evolved setups of distinct gene clusters, so-called polysaccharide utilization loci (PULs) [14, 15]. PULs target specific glycans, and encode setups of carbohydrate-active enzymes (CAZymes), sugar capture (SusD-like) and transport (SusC-like) proteins, as well as sensory and regulatory proteins. PULs targeting a variety of plant glycans have been characterized to date [4, 16–18], and by mining genomic sequences for conserved SusC/SusD-like sequences, putative PULs are now automatically predicted and collected in the PULDB database (http://www.cazy.org/PULDB/; [19]). Functional prediction of these putative PULs by sequence homology can be a quick and useful tool allowing insight into putative substrate specificity. However, as many CAZyme families are polyspecific (i.e. contain enzymes acting on various glycans), experimental data is necessary to verify the functions of PUL-encoded proteins. Recently, two xylan-targeting PULs from the gut bacterium *Bacteroides ovatus* were characterized (*Bo*XylL and *Bo*XylS) and demonstrate how a plethora of enzymes is used in the deconstruction of both AX and GAX [4]. While the majority of the encoded glycoside hydrolases (GHs) were biochemically characterized, the possible role of the only predicted carbohydrate esterase (CE), in the *Bo*XylL locus, was not investigated [4].

The predicted CE in the *B. ovatus* XylL comprises two distinct catalytic domains from carbohydrate esterase families 6 and 1 (CE6 and CE1, respectively, in N-terminus to C-terminus order). To date, all members of the CE6 family are characterized as acetyl xylan esterases in the carbohydrate-active enzymes database (CAZy, http://www.cazy.org). Conversely, members of the CE1 family display various catalytic activities, e.g. acetyl xylan esterase, feruloyl esterase (FAE), and transferase activities. The fact that the predicted CE in the xylan-targeting *Bo*XylL contains both a CE6 and a CE1 domain suggests that the enzyme could have a dual role to cleave both feruloyl- and acetyl groups [20]. An enzyme with a highly similar architecture was also found in an uncharacterized PUL encoded by the distantly related soil bacterium *Flavobacterium johnsoniae*, indicating that such CE6–CE1 fusions may have important roles in xylan depolymerization in the Bacteroidetes phylum.

Enzymes comprised of several distinct catalytic domains are typically referred to as multidomain or multifunctional enzymes. Herein we refer to such enzymes as multicatalytic, to distinguish them from enzymes composed of, for instance, one catalytic domain and one or more non-catalytic domains (e.g. carbohydrate-binding modules; CBMs), or multi/poly-specific enzymes exhibiting substrate promiscuity. Multicatalytic GH enzymes have previously been shown to be highly efficient catalysts for recalcitrant biomass degradation due to their physically linked domains with complementary functions, e.g. the cellulolytic CelA from *Caldicellulosiruptor bescii* (GH9 and GH48 cellulolytic domains; 3 CBM9 domains) and the chitinolytic ChiA from *F. johnsoniae* (two GH18 chitinases) [21, 22]. How physically connected CE domains affect their ability to complement *endo*-acting polysaccharide-hydrolysing enzymes in biomass depolymerization has to the best of our knowledge not been explored yet.

Here we investigated the role in biomass degradation of two multicatalytic enzymes from the xylanolytic gut bacterium *B. ovatus* and the soil bacterium *F. johnsoniae*, each comprising one CE6 and one CE1 domain. The enzymes were studied both in their native full-length forms as well as in truncated single catalytic domain constructs. The different catalytic domains exhibited distinct activities for different xylan substitutions and were able to substantially boost the hydrolytic action of an *endo*-acting xylanase on recalcitrant biomass. This effect was especially pronounced in the enzyme from *B. ovatus*.

## Results

### Identification of PULs encoding multicatalytic carbohydrate esterases

**Fig. 2:**
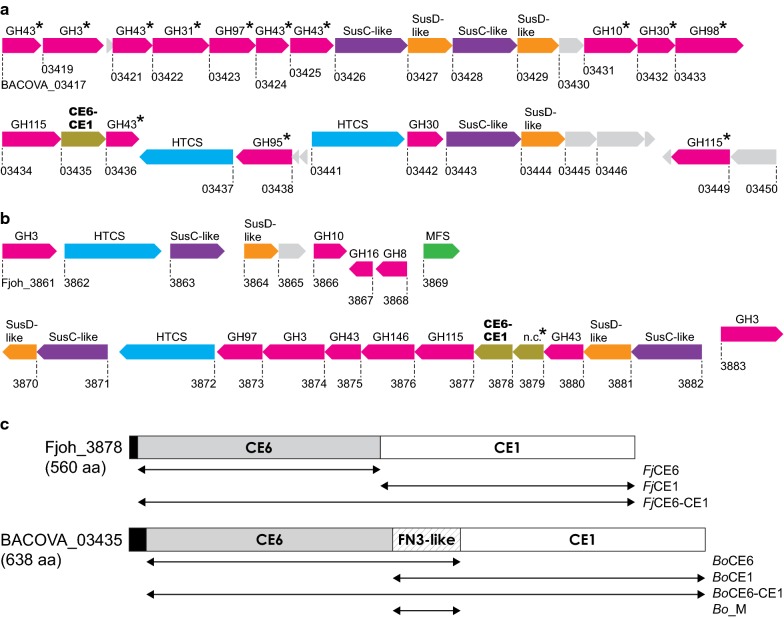
Overview of PULs encoding the studied multicatalytic esterases and the enzyme architectures. Genomic maps of the PULs: **a** the *Bo*XylL of *B. ovatus* ATCC 8483 (locus tags BACOVA_3417-3450), **b** the *Fj*XylUL of *F. johnsoniae* (*Fj*XylUL-I: Fjoh_3861-3869; *Fj*XylUL-II: Fjoh_3870-3883). Functional annotations (e.g. GH family) are shown above each gene symbol and locus tags below (n.c. = not yet classified into a CAZyme family). Genes encoding GHs are shown in pink, CEs in brown, regulatory hybrid two-component systems (HTCSs) in blue, SusC-like transport proteins in purple, SusD-like binding proteins in orange, major facilitator family transporters in green, and proteins of unknown function in grey. Previously characterized enzymes are marked with asterisks. **c** Enzyme architecture of *Fj*CE6–CE1 (Fjoh_3878) and *Bo*CE6–CE1 (BACOVA_03435) and truncated versions. Signal peptide sequences are marked in black

The anaerobic gut symbiont *B. ovatus* is a dominant member of the human microbiota and it has a vast capability to metabolize complex plant glycans thanks to its numerous PULs [23]. The recently characterized xylan-targeting PUL *Bo*XylL ([4]; Fig. 2a) has been shown, using both transcriptomic and biochemical data, to confer the ability of *B. ovatus* to metabolize highly complex GAX structures [4, 23]. One of the enzymes of the PUL, which has not been biochemically characterized, intriguingly comprises two distinct carbohydrate esterase domains, from families 6 and 1 (locus tag BACOVA_03435). We mined the PULDB for enzymes with a similar CE6–CE1 architecture and identified a large number of species and strains in the genera *Bacteroides*, *Flavobacterium*, and *Prevotella* encoding similarly comprised enzymes. The aerobic soil bacterium *F. johnsoniae* is distantly related to *B. ovatus* and encodes a putative xylan-targeting PUL which contains a similar putative CE6–CE1 enzyme (Fig. 2b). The *F. johnsoniae* PUL encodes putative enzymes predicted to target xylan decorations or oligosaccharides, such as several predicted *α*-l-arabinofuranosidases or *β*-xylosidases from glycoside hydrolase family 43 (GH43) and an α-glucuronidase from GH115 [24]. Curiously, this PUL (*Fj*XylUL-I) does not encode any putative *endo*-xylanases, though both a GH10 and a GH8 are found in a directly neighbouring PUL predicted by the PULDB (*Fj*XylUL-II). In order to enable xylan deconstruction, it is likely that these two predicted neighbouring PULs are in fact acting as a single xylan utilization locus and will here collectively be referred to as *Fj*XylUL. Recently, a novel acetyl xylan esterase, FjoAcXE (as of yet not belonging to a CE family), was identified in the *Fj*XylUL [25]. FjoAcXE has been shown to liberate acetyl moieties from both AX and also complex GAX oligosaccharides, but was unable to act on acetyl moieties linked to xylosyl moieties neighbouring a feruloylated side chain [25]. The multicatalytic CE6–CE1 enzyme, which is located directly downstream of FjoAcXE, possibly complements the activity of FjoAcXE by cleaving acetyl moieties near feruloyl moieties. In this proposed scenario, both the acetyl- and feruloyl groups could be cleaved simultaneously, or in short succession, by the CE6–CE1 enzyme.

In both *Fj*XylUL and *Bo*XylL, the predicted multicatalytic esterases, *Fj*CE6–CE1 and *Bo*CE6–CE1, respectively, are the only likely candidates able to target feruloyl moieties in their respective PULs. Additionally, the *Bo*CE6–CE1 enzyme is the only predicted esterase in both *Bo*XylL and *Bo*XylS indicating that it serves an important biological role. Both the *Fj*CE6–CE1 and *Bo*CE6–CE1 enzymes consist of an N-terminal CE6 domain and a C-terminal CE1 domain, though the *B. ovatus* enzyme contains an additional unannotated 76 amino acid insert between the CE6 and CE1 domains (Fig. 2c). The two individual CE6 domains share 69% sequence identity, while the CE1 domains exhibit only 35% sequence identity, possibly indicating larger differences in substrate specificity for the CE1 domains compared to the CE6 counterparts. These CE domains are only distantly related (15 to 30% sequence identity) to characterized enzymes of their respective families but contain the conserved catalytic residues required for functional enzymes (Additional file 1: Figs. S1 and S2). The CE1 domains are most closely related to FAE subfamily 8, based on the classification put forth by Dilokpimol et al., such as AmFae1A from *Anaeromyces mucronatus* [26]. The CE1 domains also show similarities to sub-families 5 and 6 which, while comprising FAE activities, additionally contain members with discrete acetyl xylan esterase activity (subfamily 5), indicating the importance of determining the biochemical activity of these putatively annotated enzymes.

### Biochemical characterization of the *F. johnsoniae* CE6–CE1 enzyme

**Fig. 3:**
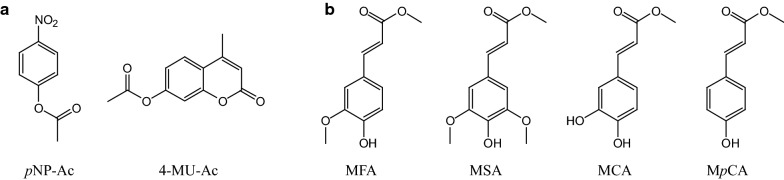
Model substrates used for the substrate specificity screen and kinetics. **a** Acetyl esterase substrates (4-methylumbelliferyl acetate, 4-MU-Ac; *p*-nitrophenyl acetate, *p*NP-Ac) and **b** feruloyl esterase substrates (methyl ferulate, MFA; methyl sinapate, MSA; methyl caffeate, MCA; methyl *p*-coumarate, M*p*CA

To investigate the function of the different domains of *Fj*CE6–CE1, the two putative CE domains were cloned and heterologously produced in *E. coli* resulting in *Fj*CE6 (N-terminal domain; 33.8 kDa) and *Fj*CE1 (C-terminal domain; 34.0 kDa) (Fig. 2c). The enzymes were assayed on a range of acetyl esterase substrates (4-methylumbelliferyl acetate, 4-MU-Ac; *p*-nitrophenyl acetate, *p*NP-Ac) and FAE substrates (methyl ferulate, MFA; methyl sinapate, MSA; methyl caffeate, MCA; methyl *p*-coumarate, M*p*CA) (Fig. 3). Kinetic parameters for the catalytic domains were determined where possible (Table 1).

**Table 1:** Kinetic parameters of single domain variants of *Bo*CE6–CE1 and *Fj*CE6–CE1, where activity could be detected

Enzyme	Substrate	*K*_M_ (mM)	*k*_cat_ (s^−1^)	*k*_cat_/*K*_M_ (s^−1^ mM^−1^)
*Fj*CE6	*p*NP-Ac	6.9 ± 2.5	57 ± 12	8.3 ± 3.5
	4-MU-Ac	0.15 ± 0.02	6.7 ± 0.2	43.4 ± 4.4
*Fj*CE1	*p*NP-Ac	0.95 ± 0.14	5.6 ± 0.3	5.9 ± 0.9
	4-MU-Ac	0.07 ± 0.01	0.61 ± 0.03	8.7 ± 1.7
	MFA	0.11 ± 0.02	2.5 ± 0.2	23.1 ± 4.5
	MSA	0.14 ± 0.02	1.8 ± 0.1	13.0 ± 2.2
	MCA	0.34 ± 0.10	0.83 ± 0.14	2.7 ± 1.0
	M*p*CA	0.39 ± 0.12	1.5 ± 0.3	3.9 ± 1.4
*Bo*CE6	*p*NP-Ac	1.8 ± 0.2	36.0 ± 2.2	20.1 ± 2.7
	4-MU-Ac	0.027 ± 0.006	1.2 ± 0.1	42.8 ± 10.0
*Bo*CE1	*p*NP-Ac	1.4 ± 0.1	0.70 ± 0.02	0.50 ± 0.04
	4-MU-Ac	0.04 ± 0.01	0.28 ± 0.01	7.1 ± 1.6
	MFA	Not saturable up to 0.3 mM		0.011 ± 3 × 10^−4^
	MSA	Not saturable up to 0.3 mM		0.043 ± 0.017

All constructs were assayed on the full range of acetyl- and feruloyl esterase substrates (*p*NP-Ac, 4-MU-Ac, MFA, MSA, MCA and M*p*CA). Results are presented as the average of three experiments with standard errors. Data were fitted to the Michaelis–Menten equation using OriginPro software. For reactions that could not be saturated with substrate, *k*_cat_/*K*_m_ was determined using linear regression

*Fj*CE6 showed activity on both acetyl esterase substrates and while the *k*_cat_ value was almost 10 times higher on *p*NP-Ac compared to 4-MU-Ac, the *K*_M_ value for the latter substrate was over 40-fold lower, resulting in a substantially higher catalytic efficiency on 4-MU-Ac compared to *p*NP-Ac (43.4 and 8.3 s^−1^ mM^−1^, respectively). No activity of *Fj*CE6 on any of the FAE substrates could be detected, which is consistent with reported enzyme activities of CE6 members. *Fj*CE1 was active on the acetyl esterase substrates, with a similar *k*_cat_/*K*_M_ value as *Fj*CE6 on *p*NP-Ac but had a fivefold lower *k*_cat_/*K*_M_ on 4-MU-Ac. *Fj*CE1 was additionally active on all FAE substrates tested and has a preference for MFA on which it displayed its highest catalytic efficiency out of all substrates (23.1 s^−1^ mM^−1^). The catalytic efficiency on MFA, a molecule closely resembling the feruloyl structures found in xylan, was almost three times higher than the highest catalytic efficiency observed on the acetyl esterase substrates. Taken together, the results strongly support the annotation of the *Fj*CE1 as a FAE.

The kinetic parameters of *Fj*CE1 on MFA were substantially better than the ones reported for two bacterial FAEs from *Streptomyces* sp. [27], which had 40-fold higher *K*_M_ values and around 60-fold lower *k*_cat_ values. Compared to the fungal FAE *An*FaeA, from *Aspergillus niger,* and the commercial enzyme E-FAERU, from a rumen microorganism, *Fj*CE1 possessed the smallest *K*_M_ value at 0.11 mM, compared to 0.78 and 0.43 mM for *An*FaeA and E-FAERU, respectively [28, 29]. However, both AnFaeA and E-FAERU possessed higher *k*_cat_ values (70.74 and 31.4 s^−1^, respectively), resulting in three to fourfold better catalytic efficiencies than those observed for *Fj*CE1. The pH optima of *Fj*CE6 and *Fj*CE1 were 8 and 6.5, respectively, when using 4-MU-Ac as substrate (Additional file 1: Fig. S3). It is noteworthy that *Fj*CE1 exhibited an activity plateau between pH 6.5 and 7.5, and still displayed 90% of its maximal activity at pH 8.0.

### Biochemical characterization of the *B. ovatus* CE6–CE1 enzyme

The CE domains of *Bo*CE6–CE1 were produced and characterized in a similar manner as *Fj*CE6–CE1. The two resulting enzymes *Bo*CE6 (N-terminal; 42.8 kDa) and *Bo*CE1 (C-terminal; 43.4 kDa) shared an overlapping middle region comprising a domain of unknown function but with a predicted fibronectin type III (FN3)-like fold, here labelled *Bo*_M (M for middle domain; Fig. 2c). Attempts to express the two CE domains excluding the *Bo*_M region resulted in highly unstable proteins not amenable for characterization.

*Bo*CE6 was, like *Fj*CE6, active on the acetyl esterase substrates. *Bo*CE6 had a higher *k*_cat_ for *p*NP-Ac than for 4-MU-Ac (36.0 and 1.2 s^−1^ mM^−1^, respectively), though it had a twofold higher catalytic efficiency on 4-MU-Ac (42.8 s^−1^ mM^−1^) due to the almost 70-fold lower *K*_M_ value (Table 1). The catalytic efficiency of *Bo*CE6 on *p*NP-Ac was double of that observed for *Fj*CE6 on the same substrate. Also, similar to *Fj*CE6, no activity on the FAE substrates could be detected for *Bo*CE6. The activity of *Bo*CE1 on the two acetyl esterase substrates was markedly lower than that of *Bo*CE6 and, in contrast to *Fj*CE1, *Bo*CE1 had no activity on M*p*CA and MCA, and only weak activity could be detected on MFA and MSA. The meagre activity on MFA and MSA, which was over three orders of magnitude lower than that observed for *Fj*CE1, might indicate that *Bo*CE1 is able to cleave natural feruloyl esters, but that the model substrates tested here are too dissimilar to an optimal substrate, such as a feruloyl moiety linked to arabinose.

To assess if the low FAE activity of *Bo*CE1 was attributable to the truncation of the enzyme, we also characterized the full-length 72.5 kDa protein. The activity on MFA and MSA was in the same order of magnitude for the full-length *Bo*CE6–CE1 as for *Bo*CE1, indicating that the low activity of the singular CE1 domain was not attributable to improper folding (data not shown). Both *Bo*CE6 and *Bo*CE1 displayed activity optima at pH 8.0 when tested on 4-MU-Ac (Additional file 1: Fig. S3).

### Investigation of the middle domain in the *B. ovatus* enzyme

As described, the 76-residue insert between the two catalytic domains of *Bo*CE6–CE1, *Bo*_M (Fig. 2c), was necessary to stabilize the *Bo*CE6 and the *Bo*CE1 proteins for characterization. Sequence analysis against the Protein Data Bank (PDB; [30]) yielded the highest sequence homology (76% query cover; 29% sequence identity) with the FN3-like domain of a putative alpha amylase from *Salmonella typhimurium* str. LT2, consisting of eight *β*-strands forming two characteristic *β*-sheets, a structure reminiscent of certain CBMs [31]. FN3-like domains found in CAZymes can act either as CBMs or play roles in protein–protein interactions [32]. To evaluate whether *Bo*_M (11.7 kDa) possessed CBM functionality we probed its ability to bind to the insoluble polysaccharides ivory nut mannan, cellulose, birch xylan, beech xylan, and the insoluble fractions of barley *β*-glucan and potato starch (Additional file 1: Fig. S5). Despite the differences in the polysaccharide backbone and decorations, *Bo*_M was shown to weakly bind all the polysaccharides tested. The lack of any specific binding may indicate that the binding was an experimental artefact. However, control reactions using bovine serum albumin (BSA) suggest otherwise since no binding was observed between BSA and the polysaccharides (data not shown). Comparing the amino acid sequence of *Bo*_M against the a recently structurally characterized enzyme from a metagenomic waste water sludge sample containing a CE1 domain fused to a CBM48, showed only a minor similarity between *Bo*_M and the CBM48 (~ 20% identity) [33]. An alternative role for the *Bo*_M domain could be to act as a spacer, allowing for a greater flexibility of the full-length *Bo*CE6–CE1 enzyme. However, further investigations are needed to fully elucidate the physiological role of this domain.

### Xylanase hydrolysis of xylan in corn cob biomass is enhanced by multicatalytic esterases

The proposed role of esterases in biomass degrading PULs is to remove the non-carbohydrate decorations on xylan and thus enable its depolymerization by other enzymes. We chose to probe this hypothesis by studying the boosting effect of our investigated CEs on the hydrolysis of ball milled corn cob (5% w/v; rich in GAX) and Japanese beechwood biomass (5% w/v; rich in GX) by a GH11 xylanase (Xyn11A from *Neocallimastix patriciarum* [34, 35]). The xylanase Xyn11A was chosen over a GH from either the *B. ovatus* or *F. johnsoniae* PULs as we wanted to evaluate the performance of the CEs in conjunction with a generic and possibly industrially relevant xylanase. Moreover, none of the putative xylanases from the *Fj*XylUL have previously been characterized, and of the characterized xylanases from the *B. ovatus* PULs, none have been shown to be able act on both GX and more substituted GAX [4]. Apart from milling and freeze-drying, the biomass was not pretreated. Corn cob biomass harbours complex GAX [4], comprising both acetylation and feruloylated arabinose substitutions [7, 36]. Japanese beechwood is a hardwood rich in acetyl moieties, but does not contain feruloyl decorations [8, 9]. Xyn11A was used in biomass hydrolysis reactions, supplemented with either one of the full-length multicatalytic CE enzymes or their single catalytic domain versions. All esterase variants were also assayed without addition of Xyn11A, which yielded no detectable release of reducing sugars (data not shown).

**Fig. 4:**
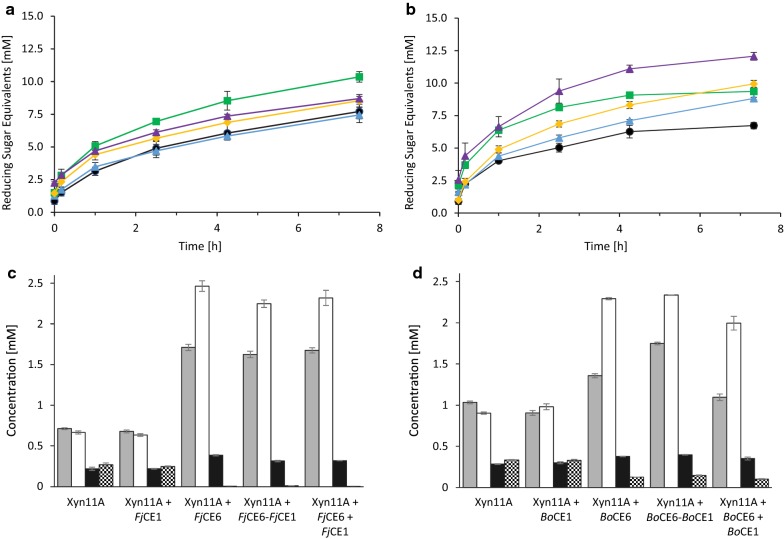
Effect of esterase supplementation on xylanase hydrolysis of corn cob biomass. Measurement of reducing sugars during reactions of the Xyn11A xylanase, alone or supplemented with the *F. johnsoniae* enzymes (**a**) or the *B. ovatus enzymes* (**b**): Xyn11A (control; black dot), Xyn11A + CE1 domain (blue triangle), Xyn11A + CE6 domain (green square), Xyn11A + equimolar mix of CE6 and CE1 (yellow dot) and Xyn11A + full-length construct (purple triangle). Xylooligosaccharide concentrations after 7.5 h incubation with Xyn11A, supplemented with the *F. johnsoniae* CE enzymes (**c**) and the *B. ovatus* enzymes (**d**): xylose (grey), xylobiose (white), xylotriose (black) and xylotetraose (checkered). Reactions with the *F. johnsoniae* constructs were performed at 25 °C, while the corresponding reactions containing *B. ovatus* constructs were carried out at 37 °C, explaining the difference in Xyn11A product release in the reactions not supplemented with esterases. Results are presented as the average of triplicate experiments with standard errors

When using the complex corn cob biomass, supplementation of Xyn11A with *Fj*CE1 did not increase the amount of reducing sugars released compared to the control reaction containing only Xyn11A (Fig. 4a). This was quite unexpected since the isolated CE1 domain was able to cleave both acetyl- and feruloyl esterase model substrates. Conversely, supplementation of Xyn11A with *Fj*CE6 yielded a 35% increase of reducing sugars released after 7.5 h. When Xyn11A was supplemented with either an equimolar mix of *Fj*CE1 and *Fj*CE6, or the full-length version *Fj*CE6–CE1, the amount of reducing sugars released after 7.5 h increased by only 10% over the control reaction without esterases. Considering the disability of the isolated CE1 domain to boost Xyn11A, the modest boosting effect seen with the combined esterases can likely be attributed to the activity of the CE6 domain. Possibly, the lower effect observed when supplementing the xylanase with either the full-length *Fj*CE6–CE1 or the equimolar mix of *Fj*CE6 and *Fj*CE1, compared to the single *Fj*CE6 could be a result of the CE1 domain blocking the access to substrate for the more efficient CE6 domain.

The potential xylanase boosting effect of the *B. ovatus* esterases (*Bo*CE1, *Bo*CE6, *Bo*CE6–CE1, and an equimolar mix of *Bo*CE1 and *Bo*CE6) were tested similarly on the corn cob biomass. Surprisingly, supplementation of Xyn11A with *Bo*CE1, which on the model substrates only exhibited trace activities, resulted in a significant boost of the xylanase reaction (Fig. 4b). During the initial phase of the reaction, almost no boosting of Xyn11A could be observed (< 10% boost after 1 h), but the effect of the *Bo*CE1 enzyme grew steadily more pronounced over time, reaching 30% after 7.5 h incubation. Similarly, supplementation of Xyn11A with *Bo*CE6 yielded a strong boost, which in contrast to the CE1 enzyme was especially pronounced during the initial phase of the reaction, with approximately 55% higher amounts of reducing sugars released after 1 h. The effect levelled off after 4 h, leading to a similar final yield of reducing sugars as the one observed for *Bo*CE1 (35% increase compared to the Xyn11A control reaction). Curiously, the equimolar mix of the *Bo*CE6 and *Bo*CE1 enzymes did not result in a strong initial boost as observed for *Bo*CE6, though the reaction did not plateau and the increase in reducing sugars after 7.5 h reached 45% compared to when Xyn11A was used alone. Finally, when the full-length *Bo*CE6–CE1 was used to supplement the xylanase, the reaction not only displayed the striking initial boost observed for *Bo*CE6, but the boosting effect continued throughout the reaction, which led to 75% more reducing sugars released after 7.5 h.

The results demonstrate that both the CE6 and CE1 domains of the *B. ovatus* enzyme contribute in supporting the xylanolytic ability of Xyn11A. Moreover, inclusion of both domains in a single enzyme resulted in a much more efficient depolymerization of the complex xylan found in the corn cob biomass. This may be due to a proximity effect, which has been shown to enhance the degradative capabilities of cellulases in cellulosomes [37] as well as fused *endo*-acting cellulolytic and chitinolytic enzymes, respectively [21, 22]. It is striking that such physical connection of complementary catalytic activities also enhances CE function, since these enzymes do not directly attack the polysaccharide backbone. A possible explanation is that fusion of these enzymatic activities enables targeting of feruloyl esters in close proximity of acetyl esters, which have been demonstrated to be problematic for the acetyl esterase FjoAcXE [25].

### Xylooligosaccharide product profiles from enzymatic corn cob hydrolysis

The xylooligosaccharide (XO; xylose (X_1_), xylobiose (X_2_), xylotriose (X_3_) and xylotetraose (X_4_)) product profiles of the xylanase reactions, with and without esterase supplementation, were assessed by high-performance anion-exchange chromatography with pulsed amperometric detection (HPAEC-PAD) after 7.5 h of incubation (Fig. 4c, d). The reactions in which Xyn11A was supplemented with either *Fj*CE1 or *Bo*CE1 did not alter XO concentrations substantially compared to the control reaction. Supplementation of Xyn11A with either *Fj*CE6, an equimolar mix of *Fj*CE1 and *Fj*CE6, or *Fj*CE6–CE1, however, doubled X_1_ and tripled X_2_ concentrations, while X_3_ concentrations only marginally increased and X_4_ was completely depleted. When Xyn11A was used together with *Bo*CE6, an equimolar mix of *Bo*CE1 and *Bo*CE6, or *Bo*CE6–CE1, the X_1_ concentrations increased by approximately 50%, X_2_ concentrations doubled, while X_3_ concentrations where slightly increased and X_4_ concentrations were halved. The consistent increase of X_1_ and X_2_ and concomitant reduction of X_3_ and X_4_ in reactions containing CE6 domains indicate that the released triose and tetraose moieties could be hydrolysed by the xylanase into mono- and disaccharides thanks to the deacetylation of the XO backbone.

Interestingly, the data showed that supplementation of Xyn11A with the *F. johnsoniae* CE enzymes yielded comparable, if not higher, X_1_–X_3_ concentrations compared to supplementation with the *B. ovatus* CE enzymes. This is in contrast with the reducing sugar analyses (Fig. 4a, b), where the total amount of reducing sugars were higher using the *B. ovatus* CE enzymes, as described above. A possible explanation for this could lie in differences in peaks corresponding to higher molecular weight XOs at later retention times in the HPAEC-PAD chromatograms. These are unfortunately not possible to quantify due to a lack of appropriate standards (Additional file 1: Fig. S4). However, qualitatively assessing the IC chromatograms showed that supplementation with the *F. johnsoniae* CE constructs (except for *Fj*CE1) led to few peaks representing higher molecular weight XO chains and no intermediate length oligosaccharides, while supplementation with the *B. ovatus* CE constructs (except for *Bo*CE1) led to numerous, but smaller peaks spanning a larger variety of XO chain lengths.

### Xylanase hydrolysis of xylan in Japanese beechwood biomass is enhanced by esterases

**Fig. 5:**
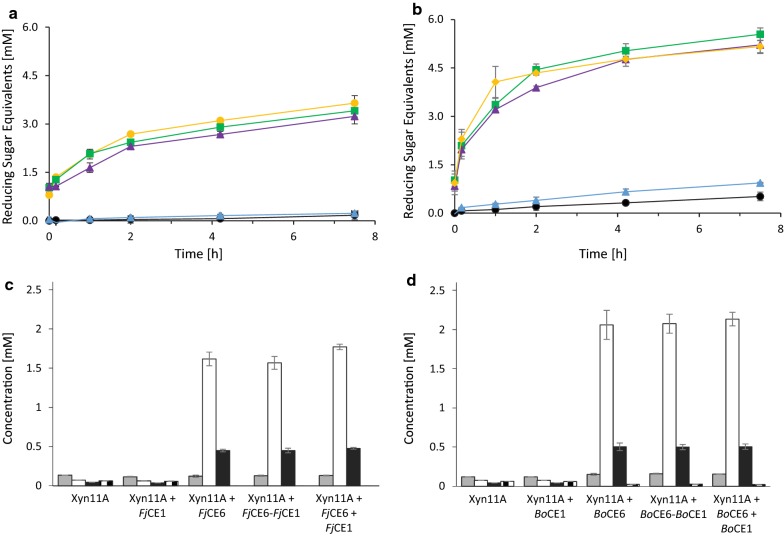
Effect of esterase supplementation on xylanase hydrolysis of Japanese beechwood biomass. Evolution of reducing sugars during supplementation of Xyn11A with the *F. johnsoniae* enzymes (**a**) and the *B. ovatus enzymes* (**b**): Xyn11A (black dot), Xyn11A + CE1 domain (blue triangle), Xyn11A + CE6 domain (green square), Xyn11A + equimolar mix of CE6 and CE1 (yellow dot) and Xyn11A + full-length construct (purple triangle). Xylooligosaccharide concentrations after 7.5 h incubation with Xyn11A, supplemented with the *F. johnsoniae* CE enzymes (**c**) and the *B. ovatus* enzymes (**d**): xylose (grey), xylobiose (white), xylotriose (black) and xylotetraose (checked). Reactions with the *F. johnsoniae* constructs were performed at 25 °C, while the corresponding reactions containing *B. ovatus* constructs were carried out at 37 °C, explaining the difference in Xyn11A product release in the reactions not supplemented with esterases. Results are presented as the average of triplicate experiments with standard errors

On Japanese beechwood the xylanase alone showed very weak activity (Fig. 5a), likely as the manifold acetyl groups shielded the xylose backbone from degradation by the xylanase. Similar to corn cob biomass, the supplementation of Xyn11A with *Fj*CE1 on Japanese beechwood biomass did not substantially increase the amount of reducing sugars released. This was expected since Japanese beechwood is not known to contain feruloyl moieties. Supplementation of Xyn11A with either *Fj*CE6, an equimolar mix of *Fj*CE6 or *Fj*CE6–CE1 increased the amount of reducing sugars released after 7.5 h by 20-fold, demonstrating an excellent boost to the activity of Xyn11A on this hardwood material. The boost seems to stem solely from the CE6 domain of the *F. johnsoniae* multicatalytic enzyme and, contrary to the experiments using the *B. ovatus* enzymes on corn cob, a benefit of a fused CE6 and CE1 domains was not observed.

Supplementation of Xyn11A with *Bo*CE1 on Japanese beechwood led only to a minor release of reducing sugars after 7.5 h, possibly as a result of the acetyl esterase activity of the enzyme (Table 1; Fig. 5b). Conversely, addition of *Bo*CE6–CE1, an equimolar mix of *Bo*CE6 and *Bo*CE1 or *Bo*CE6 drastically boosted the performance of the Xyn11A enzyme, resulting in approximately tenfold higher amounts of released reducing sugars. Similar to the *F. johnsoniae* enzymes, it appears that the enhanced xylanolytic reaction solely originates from the CE6 domain of the *B. ovatus* enzyme constructs, and a direct benefit from fused CE6 and CE1 domains was not observed on Japanese beechwood biomass. Acetate release from the Japanese beechwood biomass was examined using a commercially available kit. Samples containing CE6 domain constructs showed increased acetic acid concentrations compared to the control reaction (without CE6, 1.5 g/L acetate released; with CE6 domains present on average 3.5 g/L acetate released). Reactions containing solely CE1 domain constructs did not yield an increase in acetate concentrations (data not shown). Collectively and together with the data on the model substrates, these results substantiate the designation of the CE6 domains as acetyl xylan esterases, and the CE1 domains as feruloyl esterases.

### Xylooligosaccharide product profile on Japanese beechwood

The XO product profiles after 7.5 h of incubation on Japanese beechwood biomass for the *F. johnsoniae* (Fig. 5c) and the *B. ovatus* (Fig. 5d) enzymes show a similar pattern to the ones on corn cob biomass. Again, supplementing the Xyn11A with a CE1 domain did not alter X_1_, X_2_, X_3_ and X_4_ concentrations substantially. Supplementation of the Xyn11A with a CE6 domain (from either multicatalytic enzyme), an equimolar mix of CE1 and CE6 domains or the full-length CE6–CE1 enzyme yielded a striking increase in xylobiose and xylotriose, as well as a slight decrease in xylotetraose concentrations. For constructs containing a CE6 domain, X_2_ concentrations increased from 0.07 to 1.7 mM and 2.1 mM for *F. johnsoniae* and *B. ovatus* enzymes, respectively. X_3_ concentrations increased from 0.05 to 0.45 mM and 0.50 mM for *F. johnsoniae* and *B. ovatus* enzymes containing a CE6 domain, respectively. The similarity of XO production profiles and reducing sugar equivalent concentrations for any Xyn11A reaction supplemented with a CE6 domain, either singly or fused to a CE1 domain, supports the hypothesis that only the CE6 domain aids Xyn11A in the degradation of Japanese beechwood biomass, which is consistent with the lack of feruloyl moieties in Japanese beechwood [8, 9]. Interestingly, the released amounts of X_1_ were only minimal when the xylanase reaction was supplemented with esterases on this complex substrate. We have no clear explanation for this observation, but possibly the xylanase mainly produces X_2_ which it cannot further hydrolyse when the xylan backbone is much less substituted than the corn cob GAX.

## Discussion

Bacteria from the Bacteroidetes phylum are well-known for their carbohydrate-degradation capabilities and are found in a range of biotopes, from soils, marine and freshwater environments, to the gastrointestinal tracts of animals. Their propensity to cluster CAZymes and related proteins into PULs not only confers competitive carbohydrate acquisition mechanisms for these bacteria, but also enables targeted enzyme discovery approaches for finding new functionalities towards complex glycans [18]. Here, we investigated PUL-encoded esterases exhibiting novel architectures with multiple catalytic domains (multicatalytic enzymes) which could aid in the enzymatic degradation of plant biomass.

Both CE1 domains of *F. johnsoniae* (*Fj*CE1) and *B. ovatus* (*Bo*CE1) are likely acting as FAEs in their respective encoding organisms. *Fj*CE1 was active on all model FAE substrates and, similar to previous reports of other FAEs [38, 39], the highest activity amongst all FAE model substrates was observed on MFA, which out of the substrates most closely resembles the ferulic acid ester links found in xylan. While *Bo*CE1 only exhibited trace activity on two of the FAE model substrates (MFA and MSA), it is possible that the enzyme might perform better on a model substrate which is more similar to the structures found in xylan, such as 5-*O*-*trans*-feruloyl-l-arabinofuranose, as previously reported for an FAE from the fungus *Pleurotus sapidus* [38, 40]. The FAE family is very diverse with the CE1 grouping currently representing only a small sub-section (sub-families 5 and 6 based on the proposed classification by Dilokpimol et al. [26]). Only a few protein structures of FAE enzymes have been determined thus far, which when considering the family’s diversity makes structure and function predictions difficult. This could explain why *Bo*CE1 and *Fj*CE1 performed strikingly different on the model substrates even though they share 50% sequence identity. Furthermore, the enzymes also displayed different abilities to boost the action of the commercial xylanase Xyn11A, where *Bo*CE1 substantially facilitated an increase in reducing sugars release on corn cob biomass which, in contrast, *Fj*CE1 was unable to do.

Previously, an important loop region that governs the specificity of CE1 enzymes towards single feruloyl moieties, and not diferulate esters, by capping the enzyme’s active site cleft was identified in the fungal AmFae1A from *A. mucronatus* [41]. Both *Bo*CE1 and *Fj*CE1 contain residues spanning the AmFae1A capping loop which could also govern their respective specificities (Additional file 1: Fig. S2). However, a lack of structural information and sequence conservation amongst CE1 enzymes makes this only speculative at this point, and future structural investigations to expand our knowledge of CE1 and the FAE family as a whole are greatly needed. FAE activity may not necessarily facilitate xylanase depolymerization of GAX, as the feruloyl moieties are not directly attached to the xylan backbone, but the effect of a FAE targeting diferulate crosslinks could however reduce the overall integrity of the cell wall and thereby improve the hydrolysis by other enzymes. As such, the observation that the CE1 domain of the *B. ovatus* enzyme could boost the xylanase hydrolysis of corn cob GAX could possibly indicate an ability to target these diferulate crosslinks, while the CE1 domain of the *F. johnsoniae* enzyme possibly only targets single feruloyl moieties and thereby does not impact the action of the xylanase on corn cob biomass to the same extent.

In this study, the CE6 domains of both the *B. ovatus* and *F. johnsoniae* enzymes were shown to have acetyl esterase activity. *Fj*CE6 and *Bo*CE6 were able to aid the xylanase Xyn11A in the hydrolysis of Japanese beechwood, with the latter enhancing the hydrolysis to a greater extent (20-fold vs tenfold, respectively). As the AX of beechwood and GAX of cereals (such as corn) differ substantially in substitution patterns, acetyl xylan esterases likely have different capabilities to address the acetyl moieties on the backbone, as for instance observed for the FjoAcXE [25]. Similarly, the two CE6 domains from *F. johnsoniae* and *B. ovatus* enzymes studied here could have varying capabilities of handling these different patterns.

Another indication for different modes of action of *Fj*CE6 and *Bo*CE6 is visible in the XO product profiles obtained from the synergy experiments with Xyn11A, where the *B. ovatus* and *F. johnsoniae* enzymes, together with the xylanase, displayed dissimilar degradation patterns of the corn cob xylan (Additional file 1: Fig. S5). When the Xyn11A xylanase was supplemented with *Fj*CE6, the equimolar mix of *Fj*CE6 and *Fj*CE1, or full-length *Fj*CE6–CE1, the main peaks in the chromatograms were short XOs (X_1_–X_4_) and very long XOs (late retention times), whereas a continuous array of XO chain lengths were produced when the xylanase was supplemented with the *Bo*CE6, an equimolar mix of *Bo*CE6 and *Bo*CE1, or *Bo*CE6–CE1. Possibly, the CE6 domain of *B. ovatus* is less restricted compared to the *F. johnsoniae* counterpart, making it able to access and cleave acetyl moieties along the whole heavily substituted xylan backbone. *Fj*CE6 might in contrast be limited to act on less substituted sites and shorter XOs liberated by the *endo*-xylanase, making the resulting products consist of short oligosaccharides and longer fragments inaccessible to both the esterase and consequently the xylanase. The FN3-like domain which is present in the *B. ovatus* enzyme between the catalytic domains could possibly also influence the ability of the enzyme to degrade xylan, either by acting as a spacer or by improving binding to the substrate, though we were unable to demonstrate any strong binding abilities in our experiments.

The synergy experiments also showed an increased boost of the hydrolytic activity of Xyn11A on corn cob biomass, when supplemented with *Bo*CE6–CE1 compared to when the catalytic domains were separated or used singly. The exact mechanism behind this intramolecular cooperation between the CE6 and the CE1 domain of the *B. ovatus* enzyme is intriguing as the complementarity of the domains is not as apparent as for instance the *endo*- and *exo*-active domains of the cellulolytic *C. bescii* CelA [22]. Possibly, a simultaneous hydrolysis of acetyl- and feruloyl moieties in close proximity provides a more accessible xylan backbone for the xylanase to act, compared to when the two activities are more randomly distributed along the substrate. Another possibility that is difficult to evaluate could rather have to do with solubility vs aggregation of the polysaccharide coupled to the action of the enzymatic activities, where simultaneous action on different decorations could reduce aggregation of the polysaccharide to neighbouring acetylated and feruloylated chains.

Multicatalytic enzymes, like the *Bo*CE6–CE1 and *Fj*CE6–CE1 investigated here, could be important tools to improve complete saccharification of biomass, and are relevant for a range of biomass-dependent applications, e.g. pulp and paper, feed and food industries.

## Conclusion

Here, we presented the characterization of two multicatalytic esterases derived from xylan-targeting PULs from bacteria thriving in highly dissimilar environments. The enzymes were capable of improving the performance of a commercially available xylanase in the degradation of industrially relevant cereal (like corn) and hardwood biomass (like beechwood). The boosting effect was most pronounced on the hardwood AX, which is likely a reflection of the complexity of the cereal GAX structure. However, corn cob degradation was also substantially improved, which is important considering that corn cob is an abundant agricultural waste material, with over 1 billion metric tons of corn produced worldwide in 2018/2019 alone (US Department of Agriculture, Oct 2019). Moreover, we demonstrated that the native architecture of especially the *B. ovatus* multicatalytic enzyme was superior to the individual domains in aiding xylanase-catalysed hydrolysis of biomass. The mechanistic basis of the increased boosting effect observed for the native full-length CE6–CE1 enzyme compared to its separate active domains is yet to be elucidated but could be a result of proximity effects as demonstrated for cellulosome architectures. Future studies of similar enzymes comprising ‘accessory’ functions to classical polysaccharide-cleaving enzymes could provide further insight into the basis for the action of these interesting enzyme architectures and their applicability in enzymatic biomass conversion.

## Methods

All chemicals were purchased from Sigma-Aldrich if not stated otherwise.

### Cloning of CE constructs

*Flavobacterium johnsoniae* UW101 (generously provided by Prof. Mark McBride, University of Wisconsin-Milwaukee) was grown overnight in 5 mL lysogeny broth medium (LB) at 30 °C and 200 rpm. Genomic DNA was extracted using a NucleoSpin^®^ Soil kit (Macherey–Nagel). *Bacteroides ovatus* ATCC 8483 (generously provided by Dr. E. Martens, University of Michigan, US) was grown overnight in 5 mL LB medium at 37 °C under anaerobic conditions using a Whitley A95 Anaerobic Workstation (Don Whitley Scientific Limited) and anaerobic gas mixture (10:10:80% of H_2_:CO_2_:N_2_). Genomic DNA of *B. ovatus* was isolated using the DNeasy blood & tissue kit (Qiagen). The CE genes were amplified by PCR from genomic DNA, excluding their signal peptide sequences (identified using SignalP; [42]), with Phusion High-Fidelity DNA Polymerase (Thermo Fisher Scientific). Forward primers included 16 bp overhangs (5′-CTTCCAGGGCCATAGT-3′) and reverse primers included 18 bp overhangs (5′-TGGTGGTGCTCGAGTCTA-3′) homologous to the targeted cloning site in pET28a-TEVc. A list of the primers used is provided in Additional file 1: Table S1. pET28a-TEV is a modified pET-28a vector containing a N-terminal His_6_ tag and a TEV protease cleavage site (generously provided by Dr. N. Koropatkin, University of Michigan). The vector was digested using NdeI and XhoI (Thermo Fisher Scientific) for 1 h at 37 °C and fused with the CE-encoding genes using the In-Fusion HD cloning kit (Takara Bio). Subsequently, 1 µL of the fusion mix was added to 50 µL of competent *E. coli* stellar cells (Takara Bio), which were transformed by electroporation using a MicroPulser™ (BIO-RAD). The transformants were rescued for 1 h in LB at 37 °C and plated on LB Agar plates (2% w/v agar; 50 µg/mL kanamycin) for selection and incubated overnight at 37 °C. Single colonies were picked and cultured in 5 mL LB + 50 µg/mL kanamycin for 8 h and their plasmids were extracted (GeneJET Plasmid Miniprep kit; Thermo Fisher Scientific). 1 µL of isolated plasmid (150 ng/µL) was used to transform 50 µL of chemically competent *E. coli* BL21(DE3) (Sigma-Aldrich) using heat shock transformation. Correct plasmid constructions were confirmed by DNA sequencing (Eurofins genomics).

### Protein production and purification

Proteins were produced in 1 L cell culture volumes. The cells were induced with 0.2 mM isopropyl-β-d-1-thiogalactopyranoside (IPTG) at mid-log phase, harvested by centrifugation and disrupted by sonication. The proteins were separated from cell debris by centrifugation. The recombinant proteins were purified using immobilized metal ion affinity chromatography and one-step elution with imidazole similar to as described in Bååth et al. [43]. *Fj*CE6 and *Fj*CE6–CE1 were further purified by size-exclusion chromatography with a HiPrep 26/60 Sephacryl S-300 column (GE Healthcare) using 50 mM sodium phosphate (pH 7.5). Purified proteins were washed and concentrated in buffer (50 mM tris(hydroxymethyl)aminomethane pH 8.0, 250 mM NaCl, 5% glycerol) using 10 kDa cut-off centrifugal filters units (Amicon Ultra 15, Merck-Millipore).

### Biochemical characterization

Protein size and purity was checked by sodium dodecyl sulfate polyacrylamide gel electrophoresis (SDS-PAGE) using Mini-PROTEAN^®^ TGX Stain-Free™ Gels (BIO-RAD; Additional file 1: Fig. S6). Protein concentrations were determined using a Nanodrop 2000 Spectrophotometer (Thermo Fisher Scientific) using estimated extinction coefficients and molecular weights (Benchling). pH optima were determined using 4-MU-Ac (1 mM) in 200 µL reaction volumes of 100 mM buffer (sodium citrate pH 5.0–6.5, sodium phosphate pH 6.5–8.0, bicine pH 8.0–9.0 and CHES pH 9.0–10.0; Additional file 1: Figs. S1 and S2) at 37 °C for the *B. ovatus* proteins and at 25 °C for the *F. johnsoniae* enzymes. The fluorescent product of the hydrolysis of 4-MU-Ac was measured for 5–30 min using a plate reader (FLUOstar Omega, BMG LABTECH) at 340 nm for excitation and 520 nm for emission.

#### Enzymatic assays on model substrates

All subsequent assays were performed at the enzymes’ pH optima, using the same conditions as described for the pH optima determinations. Activity on *p*NP-Ac (1 mM) was monitored for 5–30 min and product release was measured at 405 nm using a plate reader (SPECTROstar Nano, BMG LABTECH), and 4-nitrophenyl (*p*NP) was used as a standard. Feruloyl esterase activity was evaluated on methyl esters (0.25 mM) by monitoring for 5–30 min the absorbance at 340 nm in a plate reader (SPECTROstar Nano, BMG LABTECH). Mixtures of methyl esters and their corresponding acids (methyl ferulate/ferulic acid; methyl sinapate/sinapic acid; methyl *p*-coumarate/*p*-coumaric acid; methyl caffeate/caffeic acid; Apin Chemicals) were used as standards. Kinetic parameters were obtained by varying substrate concentrations (4-MU-Ac, 0.0025–3 mM; *p*NP-Ac, 0.05–10 mM; MFA, 0.0025–0.3 mM; MSA, 0.005–0.4 mM; MCA, 0.005–0.4 mM; M*p*CA, 0.01–0.4 mM) and the obtained data was fitted to the Michaelis–Menten equation using non-linear regression (OriginPro 2018b). For reactions that could not be saturated with substrate, *k*_cat_/*K*_M_ was determined using linear regression.

#### Boosting studies with xylanase

Boosting studies were performed with the commercially available *endo*-1,4-*β*-xylanase Xyn11A (CAS 9025-57-4; Megazyme), using 5% w/v corn cob or Japanese beechwood (ball milled and freeze-dried) as substrate. Reactions were incubated in a thermomixer (Eppendorf) at 1000 rpm and 37 °C for the *B. ovatus* enzymes and at 25 °C for the *F. johnsoniae* enzymes. Time point samples were immediately frozen in liquid nitrogen and kept at − 20 °C until analysis. Prior to analysis the samples were thawed on ice, clarified by centrifugation (4 °C, 2 min, 14,000 rpm), and 15 µL of sample supernatant was mixed with 100 µL of 3,5-dinitrosalicylic acid (DNSA; [44]) reagent and 85 µL of Milli-Q-water, and the reactions incubated at 80 °C for 20 min. The concentration of reducing sugar equivalents was determined by monitoring the absorbance at 575 nm and comparing the results to a xylose standard curve and control (blank) samples without any added enzymes. X_1_–X_4_ concentrations were determined using HPAEC-PAD (high-performance anion-exchange chromatography with pulsed amperometric detection) on a Dionex ICS-5000+ (Thermo Fisher Scientific) equipped with a Dionex CarboPac™ PA200 column (Thermo Fisher Scientific) [45]. Acetate release was investigated by using the RM acetic acid (RM) kit from Megazyme, according to the manufacturer’s instructions.

#### Binding studies of Bo*_M*

Assays to evaluate the ability of *Bo*_M to bind to insoluble polysaccharides were performed on the insoluble fractions of ivory nut mannan (Carbosynth), cellulose (Merck), birch xylan (Merck), beech xylan (Merck), mixed-linkage barley glucan (Megazyme) and potato starch (Merck). 2.5% w/v solutions of insoluble polysaccharides, suspended in 50 mM tris(hydroxymethyl)aminomethane pH 8.0, 250 mM NaCl, 5% glycerol, were incubated with *Bo*_M (0.1 g/L) at 37 °C for 30 min (incubation step sample). The insoluble polysaccharides were collected by centrifugation (14,000 rpm, 5 min) before being washed with buffer and incubated for an additional 10 min (wash sample). The insoluble polysaccharides were collected by centrifugation once more and washed with 8 M urea to solubilize any bound protein (elution sample). SDS-PAGE and ImageLab (Bio-Rad) were used to visualize *Bo*_M and quantify its concentration. BSA was used as control and, similar to previous reports [21], did not bind to any of the tested substrates.

## Supplementary information

**Additional file 1: Table S1.** Primers used in this study; **Figure S1.** Sequence alignment of the CE6 domains of the *B. ovatus* and *F. johnsoniae* enzymes with related proteins; **Figure S2.** Sequence alignment of the CE1 domains of the *B. ovatus* and *F. johnsoniae* enzymes with related proteins; **Figure S3.** pH-dependent activity profiles of the *F. johnsoniae* and *B. ovatus* constructs; **Figure S4.** Peak distribution of corn cob biomass hydrolysis using HPAEC-PAD; **Figure S5.** Evaluation of binding of *Bo*_M to insoluble polysaccharides using SDS-PAGE.

## Data Availability

All data generated or analysed during this study are included in this published article and its additional information files or available from the corresponding author on reasonable request.

## Abbreviations

4-MU-Ac: 4-Methylumbelliferyl acetate
AX: Arabinoxylan
CAZy: Carbohydrate-active enzyme database
CAZyme: Carbohydrate-active enzyme
CBM: Carbohydrate-binding module
CE: Carbohydrate esterase
CX: Corn bran xylan
DNSA: 3,5-Dinitrosalicylic acid
FAE: Feruloyl esterase
GAX: Glucuronoarabinoxylan
GH: Glycoside hydrolase
HPAEC-PAD: High-performance anion-exchange chromatography with pulsed amperometric detection
IPTG: Isopropyl-β-d-1-thiogalactopyranoside
LB: Lysogeny broth
MCA: Methyl caffeate
MFA: Methyl ferulate
M*p*CA: Methyl *p*-coumarate
MSA: Methyl sinapate
PDB: Protein data bank
*p*NP-Ac: *p*-Nitrophenyl acetate
PUL: Polysaccharide utilization locus
SDS-PAGE: Sodium dodecyl sulfate polyacrylamide gel electrophoresis
Sus: Starch utilization system
TYG: Tryptone yeast glucose medium
X_1–4_: Xylose, xylobiose, xylotriose, xylotetraose, respectively
XOs: Xylooligosaccharides
XylUL: Xylan utilization locus
