# Supplementary material for: Multimodular fused acetyl–feruloyl esterases from soil and gut Bacteroidetes improve xylanase depolymerization of recalcitrant biomass
Source: Biotechnol Biofuels. 2020 Mar 31;13:60. doi: 10.1186/s13068-020-01698-9 (PMC7110780; doi:10.1186/s13068-020-01698-9)
Supplement: Supplementary file 1 — Additional file 1: Table S1. Primers used in this study; Figure S1. Sequence alignment of the CE6 domains of the B. ovatus and F. johnsoniae enzymes with related proteins; Figure S2. Sequence alignment of the CE1 domains of the B. ovatus and F. johnsoniae enzymes with related proteins; Figure S3. pH-dependent activity profiles of the F. johnsoniae and B. ovatus constructs; Figure S4. Peak distribution of corn cob biomass hydrolysis using HPAEC-PAD; Figure S5. Evaluation of binding of Bo_M to insoluble polysaccharides using SDS-PAGE. [file 13068_2020_1698_MOESM1_ESM.pdf]

Additional file 1.

Multimodular fused acetyl-feruloyl esterases from soil and gut Bacteroidetes improve xylanase depolymerization of recalcitrant biomass

Cathleen Kmezik, Cyrielle Bonzom, Lisbeth Olsson, Scott Mazurkewich, Johan Larsbrink

Table S1. Primers used in this study

Species	Construct		DNA sequence
Bacteroides ovatus ATCC 8483	*Bo*CE6	F	CTTCCAGGGCCATAGTCAAGACCCTAATTTCCATATTTATCTTTGTC
		R	TGGTGGTGCTCGAGTCTACGGAATATTAGTGGATACCGGTTC
	*Bo*_M	F	CTTCCAGGGCCGATATGGATATGACTATCCCCGGGTG
		R	TGGTGGTGCTCGAGTCTAGAAGAACGTATAACTGGAAGGATCTG
	*Bo*CE1	F	CTTCCAGGGCCATAGTGGTTGTTGTCGTATGGCA
		R	TGGTGGTGCTCGAGTCTATCAGTGTTTAAATAAATGTGGTACAAATTCT
Flavobacterium johnsoniae UW101	*Fj*CE6	F	CTTCCAGGGCCATAGTATATTTACGATTCTTTTTTTAACAGCACAGAA
		R	TGGTGGTGCTCGAGTCTAATATCCTGATAGGGACAGCATTTTATC
	*Fj*CE1	F	CTTCCAGGGCCATAGTATGCTATTTAACGGCAAAGAACCT
		R	TGGTGGTGCTCGAGTCTATTACTTCTCGATAGTATAGGCAGATATGC

Primer pairs, F: forward primer; R: reverse primer. For the amplification of constructs spanning several domains (full-length or Bo_M + active domain) the forward primer of the most N-terminal domain was combined with the reverse primer of the most C-terminal domain. Overhangs appropriate for cloning into pET28a-TEVc are highlighted in grey.

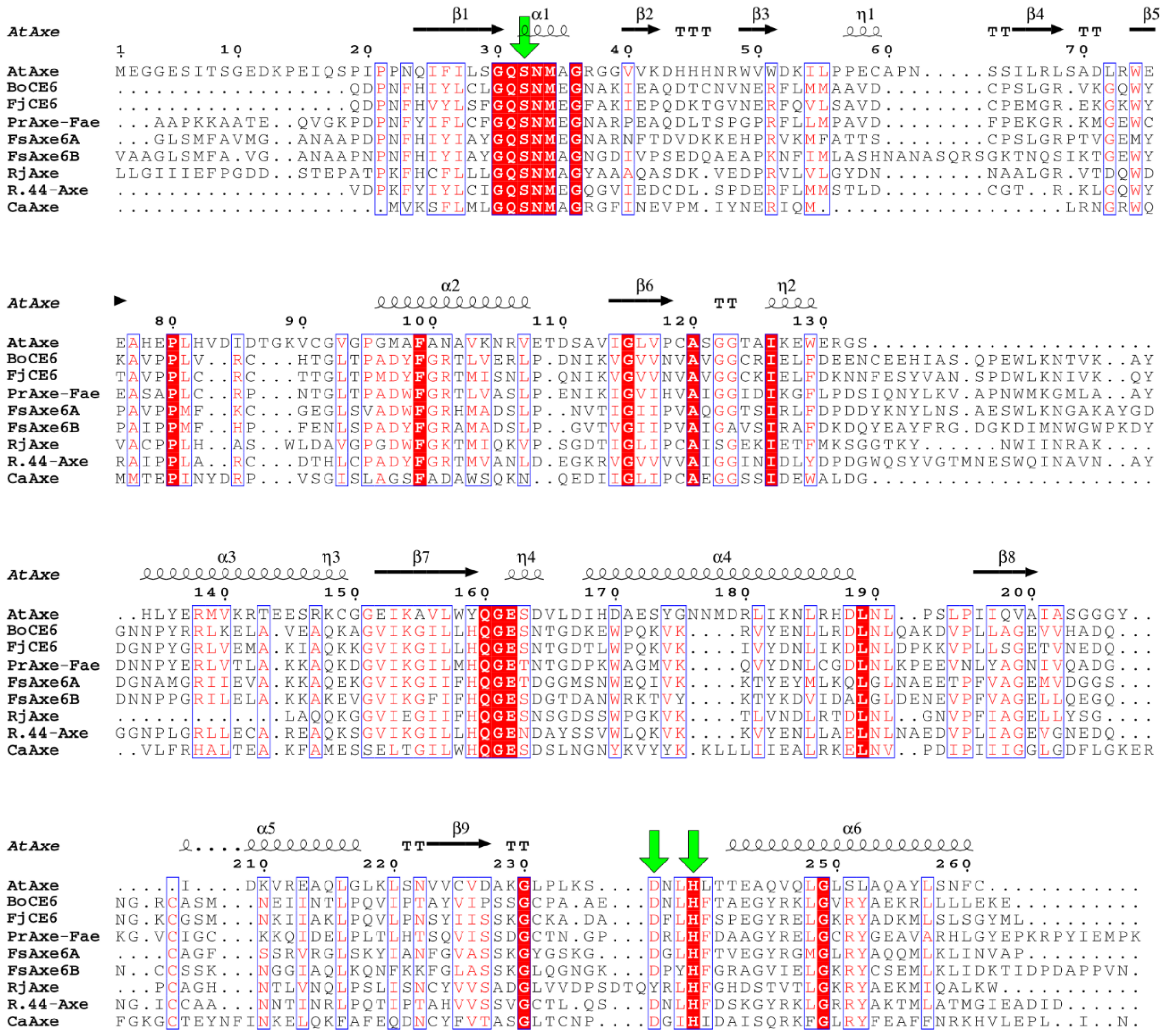

Figure S1. Sequence alignment of the CE6 domains of the *B. ovatus* and *F. johnsoniae* enzymes with related proteins. The secondary structural elements of the putative acetyl xylan esterase (Axe) from *Arabidopsis thaliana* (AtAxe; PDB accession: 2apj; [1]) are shown above the alignment. The catalytic triad identified in the AtAxe is indicated by green arrows. Additional sequences included are from *Prevotella ruminicola* (PrAxe-Fae; [2]), *Fibrobacter succinogenes* (Fs; [3,4]), *Ruminiclostridium josui* (RjAxe; [5]), a protein identified from a bovine rumen metagenome (R.44-Axe; [6,7]), and *Clostridium acetobutylicum* (CaAxe). The alignment was created in Clustal Omega [8] and visualized with Espript 3.0 [9].

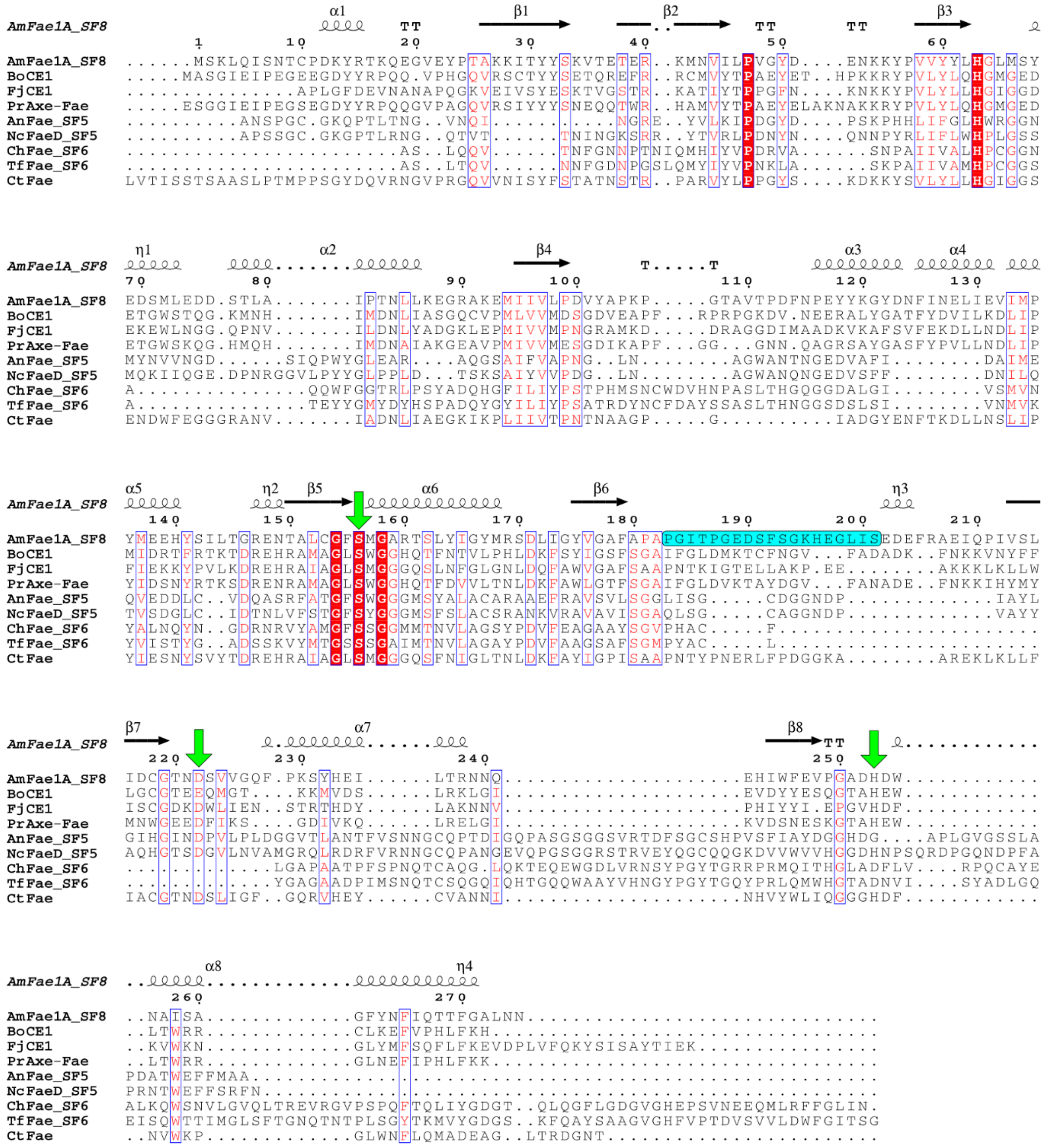

Figure S2. Sequence alignment of the CE1 domains of the *B. ovatus* and *F. johnsoniae* enzymes with related proteins. The secondary structural elements of the feruloyl esterase (Fae) A1 from *Anaeromyces mucronatus* (AmFae1A_SF8; PDB accession: 5cxu; [10]) are shown above the alignment. The catalytic triad identified in the Am_Fae1A is indicated by green arrows and its *β*-clamp region is highlighted in cyan. Additional sequences included are from *Prevotella ruminicola* (PrAxe-Fae; [2]), *Aspergilus nidulans* (AnFae_SF5; [11]), *Neurospora crassa* (NcFaeD_SF5; [12]), *Chaetomium* sp. CQ31 (ChFae_SF6; [13]), *Talaromyces funiculosus* (TfFae_SF6; [14]), and *Clostridium thermocellum* (CtFae; [15]). The sub-family (SF) number for select sequences are written after the protein name based on the classification put forth by Dilokpimol *et al.* [16]. The alignment was created in Clustal Omega [8] and visualized using Espript 3.0 [9].

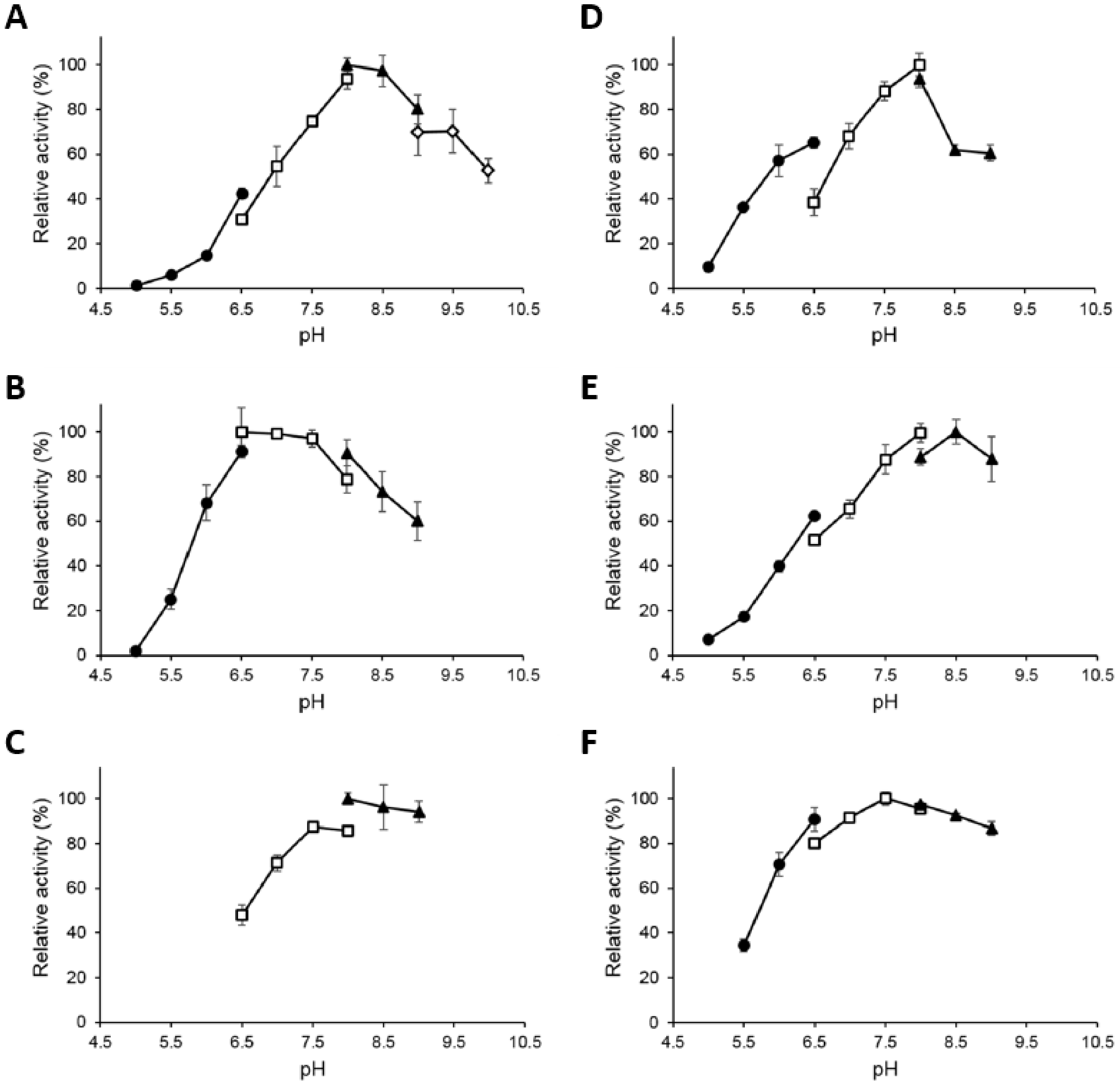

Figure S3. pH-dependent activity profiles of the *F. johnsoniae* and *B. ovatus* constructs. (**A**) *Fj*CE6, (**B**) *Fj*CE1, (**C**) *Fj*CE6-CE1, (**D**) *Bo*CE6, (**E**) *Bo*CE1 and (**F**) *Bo*CE6-CE1. pH profiles were determined by measuring activity on 4-MU-Ac (1 mM) using the buffers (100 mM): sodium citrate (closed circles), sodium phosphate (open squares), bicine (closed triangles), and CHES (open diamonds). Results are averages of three experiments, and error bars represent one standard deviation.

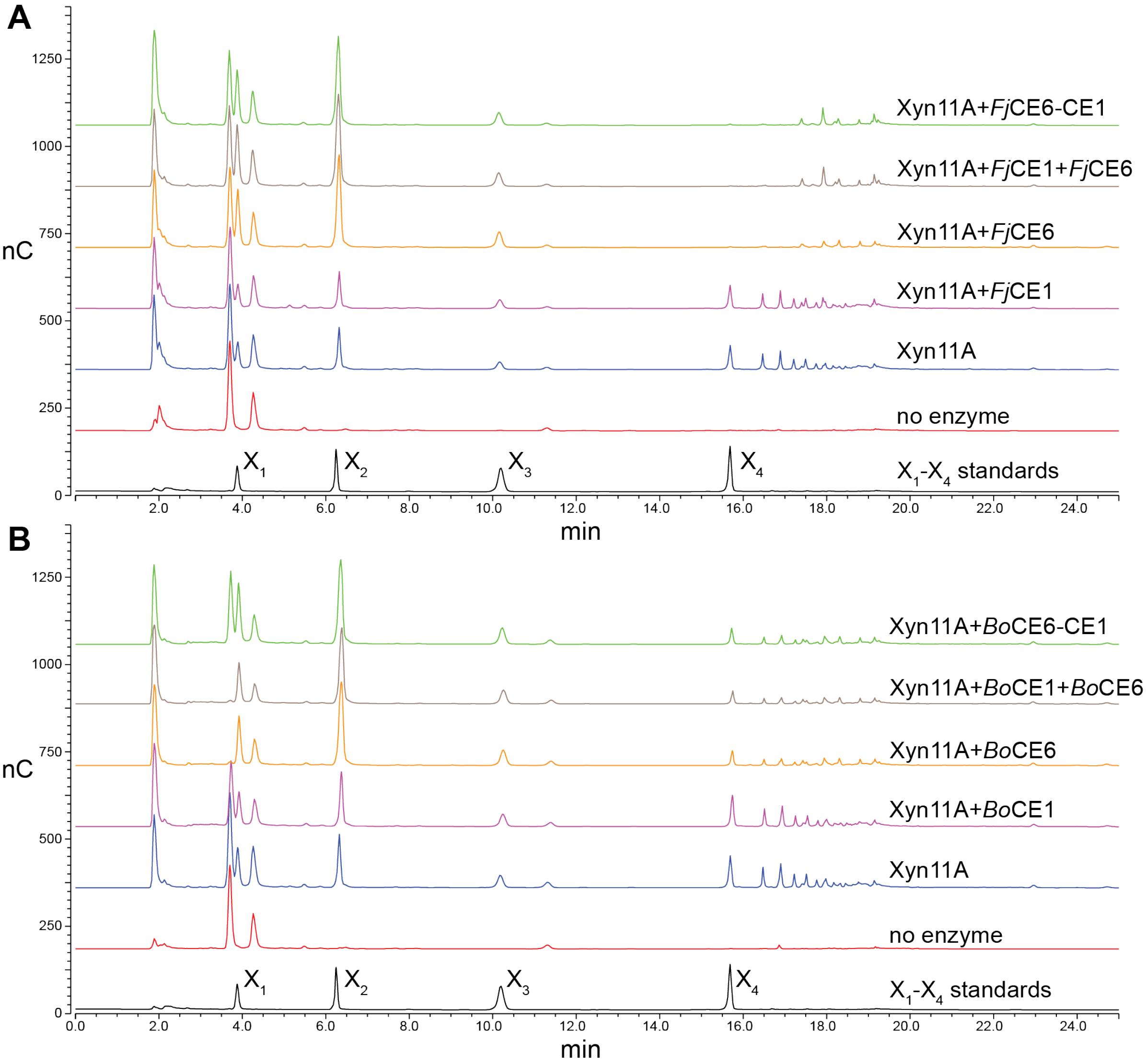

Figure S4. Peak distribution of corn cob biomass hydrolysis using HPAEC-PAD. Hydrolysis of 5% (w/v) ball milled corn cob biomass using the Xyn11A xylanase, alone or supplemented with carbohydrate esterase constructs from (**A**) *F. johnsoniae* and (**B**) *B. ovatus*. Xylose (X_1), xylobiose (X_2), xylotriose (X_3) and xylotetraose (X_4) were used as standards. Retention time is shown in minutes on the x-axis and the signal strength in nC on the y-axis. The control refers to biomass incubated without addition of any enzymes.

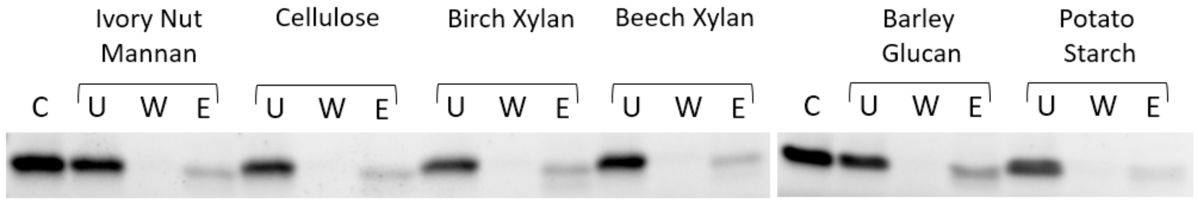

Figure S5. Evaluation of binding of *Bo*_M to insoluble polysaccharides using SDS-PAGE. C (control): *Bo*_M was incubated without addition of polysaccharides, U: unbound protein after incubation, W: protein released during wash with buffer, E: protein released during elution step with 8 M urea.

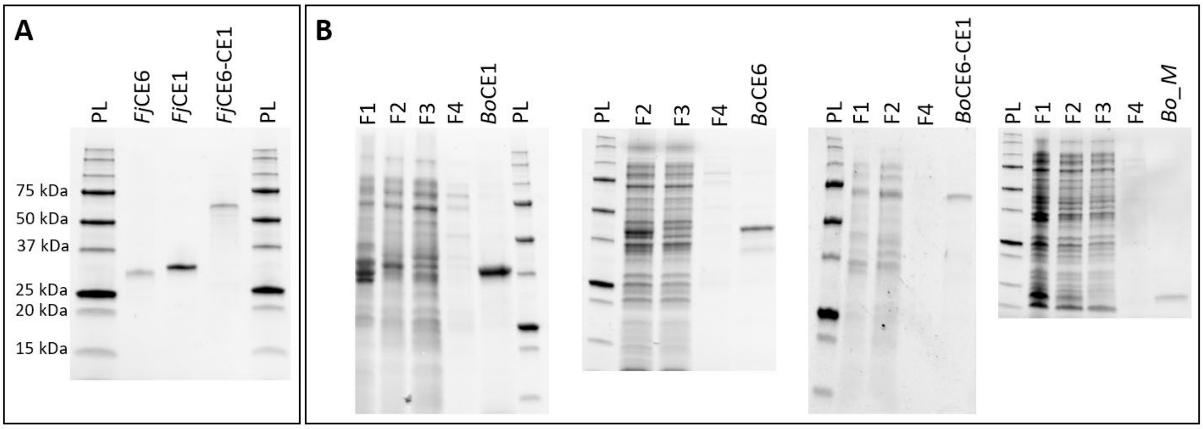

Figure S6. SDS-PAGE of enzymes originating from *F. johnsoniae* (A) and *B. ovatus* (B). The lanes containing the respective enzymes are labelled with the enzyme name. PL stands for protein ladder (Precision Plus Protein^{TM} unstained Protein Ladder from Bio-Rad). For some gels, other fractions from the purification are also shown, labelled F).

References

1. Bitto E, Bingman CA, McCoy JG, Allard STM, Wesenberg GE, Phillips Jr GN. The structure at 1.6 Å resolution of the protein product of the At4g34215 gene from *Arabidopsis thaliana*. Acta Crystallogr D Biol Crystallogr. 2005;1655–61.

2. Kabel MA, Yeoman CJ, Han Y, Dodd D, Abbas CA, Bont JAM De, et al. Biochemical characterization and relative expression levels of multiple carbohyrdrate esterases of the xylanolytic rumen bacterium *Prevotella ruminicola* 23 grown on an ester-enriched substrate. Appl Environ Microbiol. 2011;77:5671–81.

3. Kam DK, Jun H-S, Ha JK, Inglis DG, Forsberg CW. Characteristics of adjacent family 6 acetylxylan esterases from *Fibrobacter succinogenes* and the interaction with Xyn10E xylanase in the hydrolysis of acetylated xylan. Can J Microbiol. 2005;51:821–32.

4. Yoshida S, Mackie RI, Cann IKO. Biochemical and domain analyses of FSUAxe6B, a modular acetyl xylan esterase, identify a unique carbohydrate binding module in *Fibrobacter succinogenes* S85. J Bacteriol. 2010;192:483–93.

5. Wang Y, Sakka M, Yagi H, Kaneko S, Katsuzaki H, Kunitake E, et al. *Ruminiclostridium josui* Abf62A-Axe6A: A tri-functional xylanolytic enzyme exhibiting α-l-arabinofuranosidase, endoxylanase, and acetylxylan esterase activities. Enzyme Microb Technol. 2018;117:1–8.

6. López-Corés N, Reyes-Duarte D, Beloqui A, Polaina J, Ghazi I, Golyshina O V, et al. Catalytic role of conserved HQGE motif in the CE6 carbohydrate esterase family. FEBS Lett. 2007;581:4657–62.

7. Ferrer M, Golyshina O V, Chernikova TN, Khachane AN, Reyes-Duarte D, Martins Dos Santos VAP, et al. Novel hydrolase diversity retrieved from a metagenome library of bovine rumen microflora. Environ. 2005;7:1996–2010.

8. Madeira F, Park Y mi, Lee J, Buso N, Gur T, Madhusoodanan N, et al. The EMBL-EBI search and sequence analysis tools APIs in 2019. Nucleic Acids Res. 2019;47:636–41.

9. Robert X, Gouet P. Deciphering key features in protein structures with the new ENDscript server. Nucleic Acids Res. 2014;42:320–4.

10. Gruninger RJ, Cote C, Mcallister TA, Abbott DW. Contributions of a unique *β*-clamp to substrate recognition illuminates the molecular basis of exolysis in ferulic acid esterases. Biochem J. 2016;473:839–49.

11. Debeire P, Khoune P, Jeltsch J, Phalip V. Product patterns of a feruloyl esterase from *Aspergillus*

nidulans on large feruloyl-arabino-xylo-oligosaccharides from wheat bran. Bioresour Technol. 2012;119:425–8.

12. Crepin VF, Faulds CB, Connerton IF. Identification of a type-D feruloyl esterase from *Neurospora crassa*. Appl Microbiol Biotechnol. 2004;63:567–70.

13. Yang S, Tang L, Yan Q, Zhou P, Xu H, Jiang Z-Q, et al. Biochemical characteristics and gene cloning of a novel thermostable feruloyl esterase from *Chaetomium* sp. J Mol Catal B Enzym. 2013;97:328–36.

14. Kroon PA, Williamson G, Fish NM, Archer DB, Belshaw NJ. A modular esterase from *Penicillium funiculosum* which releases ferulic acid from plant cell walls and binds crystalline cellulose contains a carbohydrate binding module. Eur J Biochem. 2000;267:6740–52.

15. Schubot FD, Kataeva IA, Blum DL, Shah AK, Ljungdahl LG, Rose JP, et al. Structural basis for the substrate specificity of the feruloyl esterase domain of the cellulosomal xylanase Z from *Clostridium thermocellum*. Biochemistry. 2001;40:12524–32.

16. Dilokpimol A, Mäkelä MR, Victoria M, Pontes A, Gelber IB, Hildén KS, et al. Diversity of fungal feruloyl esterases: updated phylogenetic classification, properties, and industrial applications. Biotechnol Biofuels. 2016;9:1–18.
